# Linking gene expression to productivity to unravel long- and short-term responses of seagrasses exposed to CO_2_ in volcanic vents

**DOI:** 10.1038/srep42278

**Published:** 2017-02-13

**Authors:** Irene Olivé, João Silva, Chiara Lauritano, Monya M. Costa, Miriam Ruocco, Gabriele Procaccini, Rui Santos

**Affiliations:** 1 CCMar-Centre of Marine Sciences, ALGAE - Marine Plant Ecology Research Group. Universidade do Algarve, Campus de Gambelas, 8005-139 Faro, Portugal; 2 Stazione Zoologica Anton Dohrn, Villa Comunale, 80121, Napoli, Italy

## Abstract

Ocean acidification is a major threat for marine life but seagrasses are expected to benefit from high CO_2_. *In situ* (long-term) and transplanted (short-term) plant incubations of the seagrass *Cymodocea nodosa* were performed near and away the influence of volcanic CO_2_ vents at Vulcano Island to test the hypothesis of beneficial effects of CO_2_ on plant productivity. We relate, for the first time, the expression of photosynthetic, antioxidant and metal detoxification-related genes to net plant productivity (NPP). Results revealed a consistent pattern between gene expression and productivity indicating water origin as the main source of variability. However, the hypothesised beneficial effect of high CO_2_ around vents was not supported. We observed a consistent long- and short-term pattern of gene down-regulation and 2.5-fold NPP decrease in plants incubated in water from the vents and a generalized up-regulation and NPP increase in plants from the vent site incubated with water from the Reference site. Contrastingly, NPP of specimens experimentally exposed to a CO_2_ range significantly correlated with CO_2_ availability. The down-regulation of metal-related genes in *C. nodosa* leaves exposed to water from the venting site suggests that other factors than heavy metals, may be at play at Vulcano confounding the CO_2_ effects.

Rising atmospheric carbon dioxide (CO_2_) is increasing the concentration of dissolved inorganic carbon (DIC) in the ocean causing ocean acidification. Projections forecast that ocean acidification will continue and that by the end of the century the mean global ocean surface pH will decrease up to 0.4 units^1^, profoundly affecting marine systems^1^,^2^. Biological responses associated with ocean acidification range from changes in organismal physiology and behaviour up to changes in population structure, with major global implications for the entire ecosystem functioning and the goods and services provided^1^.

The increase in seawater DIC and CO_2_ associated with ocean acidification may favour carbon-limited species, such as seagrasses, by increasing both the passive diffusion of CO_2_ and the efficiency of RuBisCO carboxylation over photorespiration^3^, lowering the energetic photosynthetic requirements and consequently increasing photosynthetic production^2^,^4^. Physiological acclimation to ocean acidification has been widely documented in seagrasses and includes changes in photosynthetic rates, metabolism, growth and survival (e.g. refs 5, 6, 7). However, experimental evidence for increased seagrass productivity as a response to elevated CO_2_ levels and ocean acidification is inconclusive, and particularly scarce over long-time scale. Indeed, recent meta-analysis did not identify significant effects of ocean acidification on seagrass photosynthesis^8^.

The modulation of gene expression plays a central role in plant plasticity and adaptation to environmental changes^9^, since physiological machinery and metabolic pathways are coordinated at the genetic level by an array of regulatory genes, which are also affected by environmental stimuli^10^. In the last decade, molecular techniques for studying gene expression have been increasingly recognized as a powerful tool for physiological research to assess the acclimation responses and adaptive potential of marine organisms to ocean acidification (see ref. 11 for a review). Gene expression can be used to assess the role that plasticity and long-term adaptation to high CO_2_ play in altering specific metabolic pathways related with the physiological response, but also the fast acclimation and reversibility capacity to short-term acute disturbances (see ref. 12 for a review). The analysis of the expression levels of targeted genes may thus provide new understanding of molecular changes that accompany alterations in physiological states^13^,^14^.

Gene expression studies in seagrasses are yet scarce^15^ and have been mainly focused on the acclimation and adaptation potential to temperature (in *Zostera* spp.^16^,^17^,^18^) and light availability (in *Zostera marina*^19^ and in *Posidonia oceanica*^20^,^21^). Only one study looked at modulation in the gene expression of seagrasses to ocean acidification, by comparing the expression of antioxidant and stress-related genes in *P. oceanica* plants growing under the long-term influence of CO_2_ vents with plants from a control site^22^. This study targeted stress-related and antioxidant genes to infer the physiological state of plants near vents, since up-regulation of antioxidant genes is a response to a higher cellular antioxidant need resulting from the formation of reactive oxygen species (ROS) associated with increased metabolism^23^. However, so far no study integrated gene expression and physiological responses of seagrasses exposed to high CO_2_.

The combination of molecular and physiological tools may allow the identification of ecologically important gene-physiology interactions and advance our insights into how organisms respond to environmental pressure at different scales, from the fast and versatile modulation of gene expression up to the integrative physiological response. Unfortunately, research on seagrass ecophysiology and ecogenomics is not quite integrated yet^24^ and a better understanding of seagrass photosynthetic plasticity and gene-regulation is needed^25^.

Responses of organisms and communities to environmental changes span across different temporal scales, from short-term acclimation to adaptation over multiple generations. Prediction of marine organisms responses to ocean acidification has been primarily inferred by investigating short-term acclimation plastic responses in relatively short-term (from hours up to few months), single generation experiments^8^,^11^. Only few experiments tested the long-term potential for gradual acclimation or adaptation^26^ revealing that trans-generational and evolutionary adaptation can partly mitigate adverse effects of ocean acidification and highlighting the necessity of long-term experimentation^11^,^27^. A major experimental limitation for evaluating species’ long-term phenotypic plasticity and adaptive potential is to replicate the temporal scale at which ocean acidification occurs^11^.

Naturally CO_2_ enriched sites, such as submarine volcanic CO_2_ vents, have been used to study the species and communities potential to face ocean acidification. The use of CO_2_ vents provides the enormous advantage of testing organisms and communities that have been exposed for long-term over multiple generations to high CO_2_^28^, which is not feasible in experimental studies. Even though the physico-chemical composition of seeping fluids in volcanic vents is not exclusively CO_2_ and varies among vents^29^, the presence of other factors that may confound the effects of CO_2_, such as heavy metals or sulphides, have been disregarded due to the low concentrations observed at the seagrass meadows^30^. Studies conducted in volcanic CO_2_ vents revealed that seagrasses have successfully adapted to live under permanently high CO_2_ levels^31^,^32^, but support for the stimulation of photosynthetic performance and productivity of seagrasses near the vents is scarce and inconclusive. So far, studies have focused on community responses by evaluating the biodiversity and richness (e.g. ref. 33), the community structure (e.g. ref. 31) or the community metabolism (e.g. ref. 34). For the species targeted in this work, the seagrass *Cymodocea nodosa*, there is evidence of an increase in chlorophyll *a* content and maximum electron transport rate but low biomass production in meadows growing in acidified areas near CO_2_ vents^34^.

The aim of this study was to evaluate the long- and short-term responses of the seagrass *Cymodocea nodosa* to high CO_2_ to test the hypothesis of beneficial effects of ocean acidification on seagrass productivity, taking advantage of the high CO_2_ environment existing near the submerged volcanic vents at Vulcano Island (Sicily, Italy).

A combination of molecular and physiological techniques was used to relate, for the first time, the expression of target genes involved in plant metabolism (i.e. photosynthetic, carbon assimilation, antioxidant and metal-related genes) to the net plant productivity (NPP) to investigate if molecular and physiological responses are coordinated. Antioxidant and metal-related genes were also targeted to evaluate the existence of a gene activation to deal with oxidative stress and heavy metal toxicity of plants near the vents in relation to plants away from the venting influence.

A set of plant incubations with *C. nodosa* specimens and water from sites under (CO_2_ site) and away (Reference site) the influence of CO_2_ vents at Vulcano Island was performed at the same depth. Differences in the gene expression and productivity between plants exposed for a long-time to high CO_2_ (CO_2_ plants) and present-day levels of CO_2_ (Reference plants) were investigated (*in situ* incubations) to assess if plasticity and long-term adaptation to high CO_2_ affected specific metabolic pathways and productivity response of *C. nodosa*. The short-term acclimation responses to CO_2_ and reversibility capacity of *C. nodosa* were assessed investigating the gene expression and productivity of plants from one site (CO_2_ or Reference) incubated with water from the other site (transplant incubations).

To validate the observed effects of high CO_2_ on productivity and evaluate the adequacy of volcanic CO_2_ vents as natural laboratories, a set of controlled experimental incubations of *C. nodosa* plants were conducted in Ria Formosa (Portugal) under a wide range of CO_2_ concentrations.

## Results

### Seawater parameters

A description of the seawater parameters and irradiance during the incubations conducted both in Vulcano (Italy) and Ria Formosa (Portugal) is given in Table 1. In Vulcano, mean pCO_2_ concentrations (and associated parameters of the DIC system) were significantly higher in the CO_2_ site than in Reference site (p < 0.05, df = 6). In accordance with descriptions from previous studies^29^,^30^,^34^ conducted in this site, seawater chemical variability near the venting site (CO_2_ site) was higher than in the Reference site, particularly regarding the inorganic carbon system. Indeed, occasional extreme high values of pCO_2_ and DIC were recorded in the CO_2_ site (Table 1).

### Vulcano Incubations

#### Gene expression

Expression stability of reference genes (RGs): Four RGs (Eukaryotic initiation factor 4A (eIF4A), Glyceraldehyde 3-phosphate dehydrogenase (GAPDH), 18S ribosomal RNA (18S) and Ubiquitin (UBI)), belonging to different functional classes, were tested for stability in the different experimental conditions (Table 2). The three algorithms applied (BestKeeper, geNorm and NormFinder) agreed in suggesting the eIF4A as the best RG (see Supplementary Tables S1, S2 and S3 in Appendix 1, Supplementary Information). However, we decided to include GAPDH as RG, indicated by geNorm, and also the 18S, the second best RG according to NormFinder and geNorm, for target gene expression normalization (Supplementary Table S4). The use of three RGs allowed for a more reliable normalization of gene expression data.

Genes of interest (GOIs) expression level: Relative expression levels of 20 GOIs involved in photosynthesis, carbon dioxide fixation and metabolic carbon assimilation pathways, antioxidant and metal responses were analysed in *C. nodosa* plants from both Reference and CO_2_ sites after *in situ* and transplanted incubations (Table 2).

**Figure 1:**
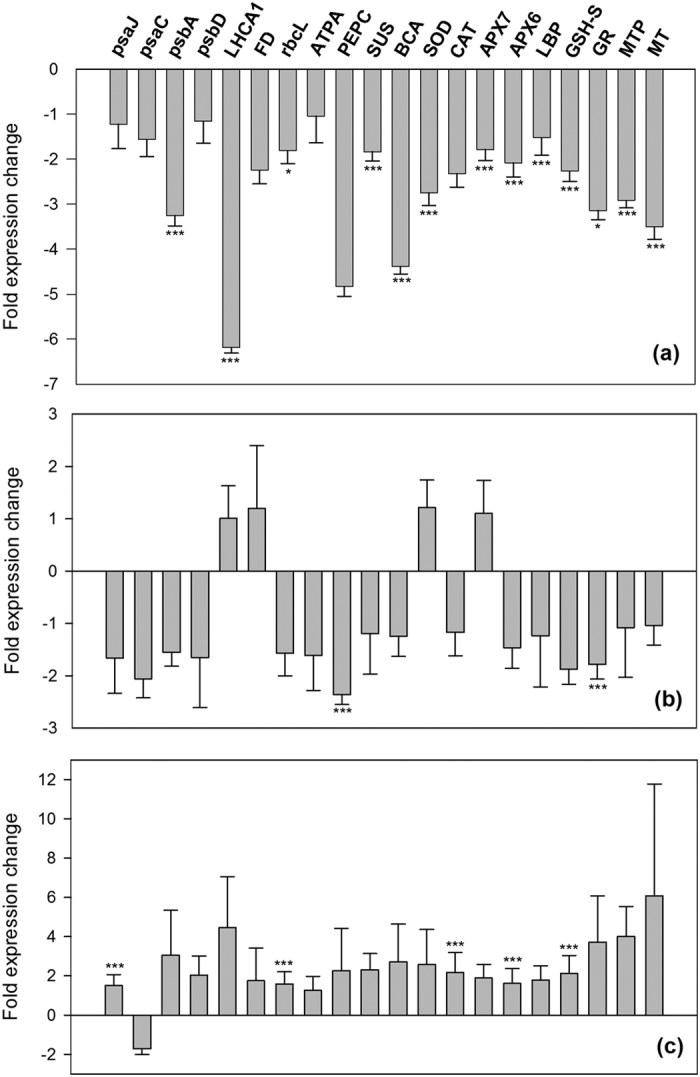
Relative expression of photosynthetic, antioxidant and metal detoxification-related genes. (**a**) Bars indicate fold expression changes of CO_2_ plants incubated in CO_2_ water with respect to Reference plants incubated in Reference water. (**b**) Bars indicate fold expression changes of Reference plants incubated in CO_2_ water with respect to Reference plants incubated in Reference water. (**c**) Bars indicate fold expression changes of CO_2_ plants incubated in Reference water with respect to CO_2_ plants incubated in CO_2_ water. Error bars represent standard error. (*) P(H1) < 0.05; (***) P(H1) < 0.001.

Major changes in gene expression patterns were observed in plants long-term growing near the vents (*in situ* incubations in CO_2_ site) with respect to plants from the Reference site (*in situ* incubations in Reference site) (Fig. 1a), as confirmed by the multivariate analysis of similarity (ANOSIM), which revealed a certain degree of separation between the two sites (Global R: 0.370, p = 0.098, Supplementary Table S5). Overall, expression levels of genes involved in light-dependent reaction of photosynthesis, carbon dioxide fixation and carbon assimilation pathways, were reduced in plants long-term growing in the CO_2_ site compared to plants from Reference site (Fig. 1a). According to univariate REST analysis, a significant down-regulation was observed for the subunit psbA of the Photosystems II, the Photosystem I Light Harvesting Complex gene 1 (LHCA1), the Beta carbonic anhydrase (BCA), and the Sucrose synthase (SUS) (*P(H1)*: 0.000), as well as the large subunit of RuBisCO (rbcL) (*P(H1)*: 0.037). The transcripts for the Ferredoxin (FD) and Phosphoenolpyruvate carboxylase (PEPC) were close to the significance level (*P(H1)*: 0.058 for both) (Supplementary Table S6). The same pattern was found for genes involved in the antioxidant response and metal detoxification (Fig. 1a). The expression of genes involved in reactive oxygen species (ROS) detoxification enzymes (antioxidant system), such as Superoxide dismutase (SOD) and Ascorbate peroxidases (APX7 and APX6), was significantly reduced, as were genes involved in glutathione metabolism (GSH-S and GR) and luminal binding protein (LBP) (*P(H1)*: 0.000). Down-regulation of metal tolerance and metal detoxification proteins (MTP and MT) was also significant (*P(H1)*: 0.000). The general down-regulation pattern observed points to an overall reduction of metabolic processes related with photosynthesis, oxidative (ROS) and metal detoxification response in plants growing near the CO_2_ vents with respect to plants from the Reference site.

When specimens of *C. nodosa* from the Reference site were incubated in water from the CO_2_ site (transplant incubation), expression of almost all the GOIs analysed was reduced (Fig. 1b) also indicating an overall reduction of metabolic processes related with photosynthesis and antioxidant response when compared to *in situ* conditions (i.e. plants from the Reference site incubated in water from the Reference site). Significant down-regulation was also detected for the enzymes Phosphoenolpyruvate carboxylase (PEPC) and Glutathione reductase (GR) (*P(H1)*: 0.000) (Supplementary Table S6).

On the contrary, when plants growing in the CO_2_ site were incubated in water from the Reference site, a clear gene up-regulation pattern was observed on the metabolic paths linked to photosynthesis as well as oxidative (ROS) and metal detoxification to acclimate to the new conditions (Fig. 1c). Although multivariate differences in gene expression were not significant (Global R: 0.370, p = 0.200, Supplementary Table S5), univariate REST analysis revealed a significant up-regulation of the photosynthesis-related genes psaJ, and rbcL, as well as the antioxidant-related genes CAT, APX6 and GSH-S (*P(H1)*: 0.000) (Fig. 1c). Several GOIs analysed (psbA, L, LHCA1, SUS, BCA, APX7,GR, MTP) were also close to the significance level (*P(H1)* < 0.1) (Supplementary Table S6).

#### Net plant productivity

**Figure 2:**
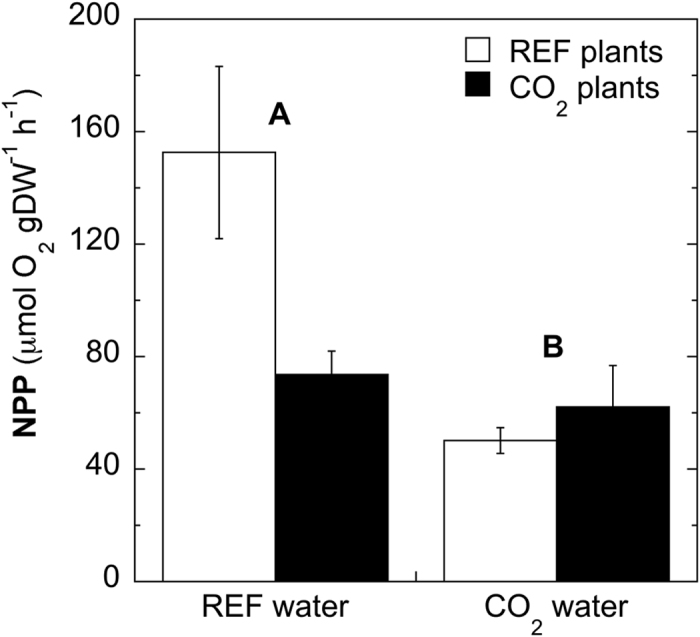
Net plan productivity (NPP) of *in situ* and transplanted incubations of *Cymodocea nodosa* plants. Letters (**A**,**B**) indicate significant differences between incubations conducted with water from the Reference and CO_2_ site. Bars represent means ± sem.

In Vulcano, the net plant productivity (NPP) of *C. nodosa* was significantly affected by the origin of the water where plants were incubated (p < 0.01). NPP was significantly lower when plants were incubated with water from the CO_2_ site, both in plants long-term growing near the CO_2_ vents (i.e. CO_2_ plants *in situ* incubations) or away from them but incubated with water from the CO_2_ site (i.e. Reference plants, REF, transplant incubations) (Fig. 2).

Plants collected from both sites did not differ significantly in their exposure to different CO_2_ concentrations in water (i.e. no significant interaction “plant origin” x “water” was detected, F_1,25_ = 2.698, p = 0.113, see ANOVA results in Supplementary Table S7).

#### Gene expression and productivity

**Figure 3:**
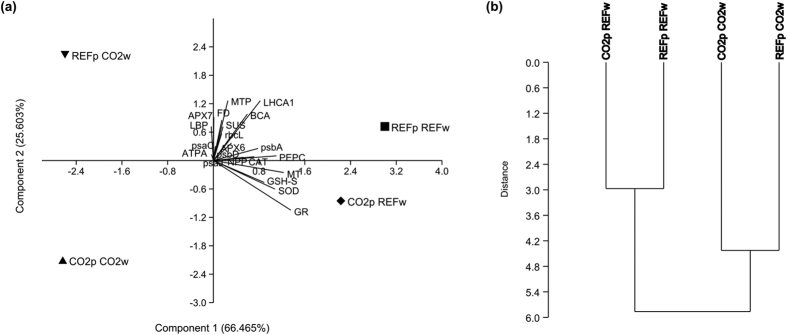
PCA (a) and Cluster analysis (b) conducted considering the combined contribution of all GOIs analysed and NPP on *in situ* and transplanted incubations of *Cymodocea nodosa* plants. REFp REFw: *in situ* incubations of Reference plants in Reference water; REFp CO2w: transplant incubations of Reference plants in CO_2_ water; CO2p CO2w: *in situ* incubations of CO_2_ plants in CO_2_ water; CO2p REFw: transplant incubations of CO_2_ plants in Reference water.

A PCA and cluster analysis and a correlation analysis were performed to link gene expression and productivity results. The PCA and Cluster analysis, conducted considering the combined contribution of all GOIs analysed and NPP, clearly highlighted two groups (PCA component 1 explaining 66.5% of the total variance) defined by the origin of the water where plants were incubated (i.e. water from Reference *vs.* CO_2_ site) (Fig. 3). These two groups matched the patterns described in Figs 1 and 2. A general up-regulation of photosynthetic, antioxidant and metal detoxification-related genes and higher NPP was found in plants long-term growing in the Reference site (*in situ* incubations) and plants from CO_2_ site incubated in water from the Reference site (transplant incubations). Reversely, plants long-term growing in the CO_2_ site (*in situ* incubations) and plants from the Reference site incubated in water from the CO_2_ site (transplant incubations) showed a generalized gene down-regulation and lower NPP. The biplot in the PCA identify GR, MT, PEPC, SOD, GSH-S, LHCA1 and psbA as the genes most contributing to this separation (for detailed information on the contribution of all GOIs and NPP see Supplementary Table S8).

Direct correlations were found between gene expression and productivity (Spearman’s rank correlation). Significant correlations were detected between NPP and expression of photosynthetic (psaJ, psbA, LHCA1 ρ = 0.49, p < 0.01), and carbon metabolism-related genes (PEPC, ρ = 0.59, p < 0.001) as well as antioxidant-related genes (SOD, GR ρ = 0.59, p < 0.001 and GSH.S ρ = 0.39, p < 0.05) (Supplementary Table S9).

### Ria Formosa Incubations

**Figure 4:**
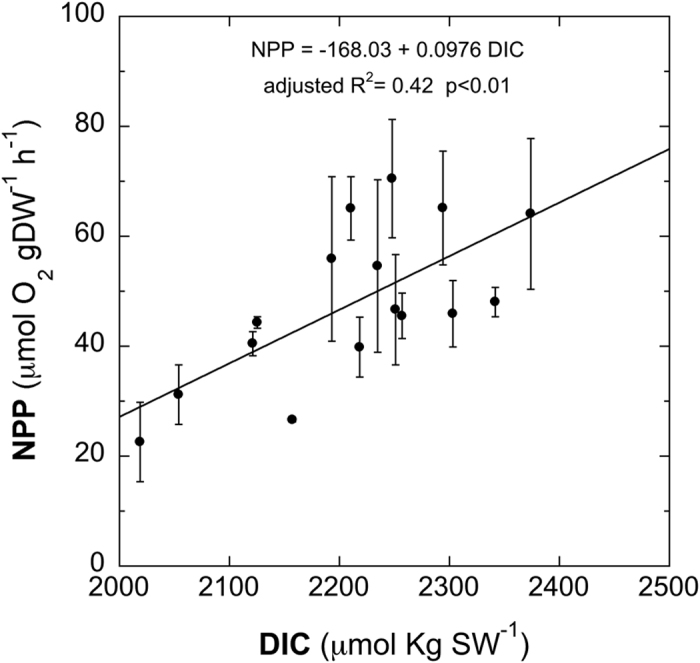
Net plan productivity (NPP) *vs.* dissolved inorganic carbon (DIC) on incubations of *Cymodocea nodosa* conducted in Ria Formosa. Dots represent mean ± sem. Linear regression equation, adjusted R^2^ and p-value are also indicated.

In Ria Formosa, where there are no volcanic vents, a set of *in situ* incubations of *C. nodosa* plants were performed under the same physico-chemical conditions with CO_2_ levels experimentally controlled (Table 1). NPP of *C. nodosa* plants in the Ria Formosa incubations showed a significant positive correlation with DIC (adjusted R^2^ = 0.42, p < 0.01) (Fig. 4).

## Discussion

Our results showed that both the net plant productivity (NPP) and the gene expression of *C. nodosa* were significantly lower in plants incubated in CO_2_–rich water from Vulcano CO_2_ vents when compared to Reference water. Plants living in the venting site showed a general reduction in the expression of genes involved in various metabolic processes, particularly those related with the light-dependent reactions of photosynthesis, carbon fixation and metabolic carbon assimilation when compared to plants from the Reference site. The same pattern was observed for genes involved in the oxidative stress antioxidant response and metal detoxification. This generalized gene down-regulation was paralleled by a reduction in plant metabolism, which resulted in 2.5 fold decrease of NPP in plants naturally growing under acidified water near the vents. Broad-scale decreases in the expression of genes related to key cellular processes, including metabolism and stress response, affecting physiological processes have been already described in different marine taxa in response to ocean acidification (ref. 35 and references therein). During long-term CO_2_ enrichment, down-regulation of RuBisCo has been described in C_3_ plants as a photosynthetic acclimation response (ref. 25 and references therein). A reduction in global metabolism has been also described in many marine organisms under high CO_2_ conditions^8^. A general down-regulation of photosynthesis-related genes has been described in seagrasses of the genus *Zostera* in response to temperature increases and global warming^17^, but current knowledge on seagrasses gene expression patterns under high CO_2_ conditions is still very limited. The only other available study described variable patterns on expression of stress-related genes in the seagrass *Posidonia oceanica* growing near the volcanic CO_2_ vents of Panarea and Ischia Islands (Italy)^22^. In agreement with our results, these authors reported down-regulation of several metal-detoxification and antioxidant genes and suggested there was no need for activation of these metabolic defence mechanisms due to the low heavy metal-stress and oxidative damage. Seagrass species show many convergent adaptive features to allow life in the marine environment (see refs 36 and 37), however different lineages can have different response to specific biotic and abiotic factors. We have analysed and identified reference genes (RGs) that are stable in *C. nodosa* exposed to volcanic CO_2_ vents and the expression level of the genes of interest (GOIs). The pioneer information reported here can represent a baseline for understanding adaptive features and stress responses in this species and in the Cymodoceaceae family in general.

The short-term acclimation responses of *C. nodosa* to high CO_2_, evaluated through the transplant incubations, agreed with the molecular and productivity long-term responses measured *in situ*. Reference plants incubated in water from the CO_2_ site experienced a generalized, drastic down-regulation in the gene expression of several photosynthetic, antioxidant and metal-related genes that globally reduced plant metabolism and resulted in a significant NPP decrease. On the contrary, plants from CO_2_ site incubated in Reference water significantly up-regulated the expression of those genes and moderately increased NPP.

Globally, we identified a coordinated gene expression pattern among photosynthetic antioxidant and metal-detoxification genes in all incubations conducted in Vulcano. We also detected a fast response of gene expression over physiology and global metabolism in *C. nodosa* leading to significant differences in plant productivity when incubated in water with different CO_2_ concentrations. Significant positive correlations were detected between gene expression and productivity for specific genes encoding proteins related with the efficiency of the photosynthetic process, such as the Photosystem I reaction center (psaJ) and the Photosystem II protein D1 (psbA), the Photosystem I light harvesting complex (LHCA1), and the carbon fixation (Phosphoenolpyruvate carboxylase, PEPC). Significant correlations were also detected between oxidative stress-related genes (Superoxide dismutase, SOD, Glutathione reductase, GR, and Glutathione synthase, GSH-S) and productivity. These results support the use of molecular tools as early indicators of potential stress or physiological readjustments in response to environmental change^38^ since metabolic pathways and physiological machinery are primarily gene-coordinated^10^. They may thus provide earliest evidence of organism responses before than morphological and physiological indicators^39^. Work is still needed to untangle the scaling-up control from genes, over protein expression and biochemistry up to physiology in seagrasses, their energetic implications and the metabolic consequences.

The contrasting response pattern recorded in Vulcano between plants long-term growing at each site (*in situ* incubations) could be attributed to local adaptation and differential selection of specific genotypes under acidified conditions^40^. However, preliminary results of a population genetic analysis carried out in Vulcano using microsatellite markers, at the same time and sampling sites than the present study, showed no genetic differentiation and high gene flow between plants from CO_2_ and Reference sites (Silva *et al. in prep*.). Consequently, results point to a low contribution of local adaptation to the observed differences between plants from the two sites. Organisms may also be able to cope with conditions found within the CO_2_ vents through plastic responses at different levels, from molecular (i.e. gene expression) to physiological, morphological and behavioural, without genotypic changes occurring through selection^41^. The large photosynthetic plasticity attributed to *C. nodosa*, in terms of photosynthetic response capacity^42^,^43^, could thus explain the differences found between specimens in Vulcano sites and the rapid acclimation mechanisms triggered during the transplant incubations.

The observed decrease of *C. nodosa* productivity under the influence of the high CO_2_ vents in Vulcano island appears to contradict the common assumption that seagrasses will increase their productivity in a foreseen high CO_2_ world. Contrastingly, the pattern obtained in Ria Formosa incubations, where CO_2_ availability was the only factor tested, significantly correlated with DIC availability. This latter pattern supports the hypothesis that seagrasses are C-limited at current CO_2_ concentrations^4^,^32^,^44^ and that they may benefit from higher DIC availability, enhancing photosynthetic rates and productivity if no other limiting factors, such as irradiance, nutrients or temperature, are present^7^. The photosynthetic plasticity attributed to *C. nodosa*^42^,^43^ could once more explain, via acclimation to local conditions, the differences found between specimens from distant locations long-term growing under different environmental pressure (i.e. high CO_2_ exposure in Vulcano *vs.* present day CO_2_ exposure (*ca.* 380 μmol pCO_2_) in Ria Formosa) when exposed to high CO_2_. However, the sharp NPP decrease observed in *C. nodosa* growing at present day CO_2_ levels in Vulcano (Reference plants) when incubated in CO_2_-rich seawater (i.e. transplants) is difficult to justify, particularly when considering the photosynthetic carbon-limited physiology of this species^44^,^45^ and the significant correlation of NPP to CO_2_ showed by Ria Formosa plants short-term exposed to a CO_2_ range.

The PCA analysis revealed a consistent pattern between gene expression and productivity indicating water origin as the main source of variability among all the incubations conducted in Vulcano. Results point to the possibility that other environmental factors related to volcanic vent emissions may correlate with the high CO_2_ in Vulcano^46^. Since plants from both CO_2_ and Reference sites in Vulcano were growing and incubated under similar environmental conditions (i.e. irradiance, temperature, salinity (Table 1) and nutrients^47^) only differing in their exposure to the venting seeping fluid, its specific physico-chemical composition may be the cause of the particular response of *C. nodosa*.

Submarine CO_2_ vents share the common trait of CO_2_ being the main gas emitted. However, the particular physico-chemical composition of seeping fluids is site specific and varies widely among vents^29^. The seeping fluid in Vulcano has been characterized as a CO_2_ source (97–99%) with sulphide (H_2_S) concentrations of about 270 μM^48^ and minor concentrations of trace elements and toxic metals (e.g. Cd, Co, Cr, Cu, Mn)^46^. Negative and toxic effects of H_2_S and heavy metals on the photosynthetic apparatus, metabolism and survival of marine organisms, including seagrasses, have been previously described^49^,^50^. The down-regulation of metal detoxification genes (MTP and MT) recorded in *C. nodosa* leaves when incubated in water from CO_2_ site suggests no significant damage associated with heavy metals. However, attenuation, and even inhibition, of cytochromes functioning has been reported at concentrations of H_2_S as low as 100 μM compromising the photosynthetic output of photosystems^51^,^52^. This is a likely explanation for the significant down-regulation detected in the photosynthesis-related genes, particularly in the photosystems I and II, and the lower productivity of *C. nodosa* plants incubated with water from the venting site in Vulcano. Unfortunately, no data on seawater sulphide or metal concentration during the experimental period are available. At present, a major knowledge gap exists regarding the combined effects of high CO_2_ and other drivers associated with Global Climate Change on marine benthic communities, including those dominated by seagrasses. The particular chemical composition of seeping fluids at each venting site may recreate different foreseen multi-drivers scenarios associated with Climate Change (i.e. hypoxia, high temperature, sulphide and metal presence, anthropogenic pressure)^1^.

In conclusion, we related, for the first time, gene expression with net plant productivity in the seagrass species, *Cymodocea nodosa,* exposed to high CO_2_ in volcanic CO_2_ vents. We found that the gene expression and productivity responses of *C. nodosa* to high CO_2_ at Vulcano vents were coupled indicating water origin as the main source of variability among all the incubations. However, the hypothesis that high CO_2_ environment near the vents is beneficial to plant productivity was not supported. The consistent and unexpected long- and short-term pattern of gene down-regulation and NPP decrease observed in *C. nodosa* when incubated with water from the vent site suggests that environmental factors, other than increased inorganic carbon availability, may be at a play affecting photosynthetic metabolism and decreasing productivity. The down-regulation of metal detoxification genes suggests no significant damage associated with heavy metals. Further research is needed to clarify if sulphide or other components of seeping fluids may explain our observations. Natural submarine CO_2_ vents provide unique experimental conditions to evaluate the acclimation and adaptive potential of species and communities to scenarios where ocean acidification occurs in combination with other environmental factors.

## Material and Methods

### Site Description

This study was conducted in May 2013 in Vulcano Island (Sicily, Italy) where submarine gas emissions date from late 18^th^ century^53^. The experimental site was located in Levante Bay, a shallow bay under the influence of a submarine volcanic vent^46^. A pH gradient is observed along the coast, with the lowest values near the venting emission points (pH 5.65) increasing to normal values (pH 8.1) at about 400 m away from the vents^30^. Fluctuations in DIC and pH along this gradient have been described depending on wind direction and consequent water mass movement^30^.

We selected two sites, characterized by different pH regimes, where the seagrass *Cymodocea nodosa* grows^30^,^34^,^46^. The low pH/high CO_2_ site (CO_2_) is located approximately 200 m from the vent at 2 m depth and 50 m from the shore. The Reference site (REF), away from the vent influence, is located approximately 450 m from the vent at 2.5 m deep and 40 m from the shore.

In order to test the productivity results obtained in Vulcano Island, we conducted a set of incubations at an independent control site. Specimens of *C. nodosa* growing in Ria Formosa (Portugal), a lagoon system with no venting influence, were incubated following the same methodological approach used in Vulcano, i.e. *in situ* NPP estimates through O_2_ determination in the same incubation chambers used in Vulcano.

### Seawater parameters

During the incubations, underwater irradiance (Licor Li-192SA) above the incubation chambers, salinity (VWR CO310), temperature (CheckTemp1, Roth), and pH (Thermo Scientific Orion) were monitored in both Vulcano (Italy) and Ria Formosa (Portugal). Alkalinity, for characterization of the DIC system, was measured by final point potentiometric titration and accuracy (~5 μmol·Kg SW^−1^) was checked using CRM batch #126 (Dickson laboratory, Scripps). DIC system parameters were calculated with the CO2SYS program^54^ using the dissociation constants of Mehrbach *et al*. (1973) refitted by Dickson and Millero (1987) (references included in the CO2SYS).

### Plant incubations

*Cymodocea nodosa* plants, each unit consisting of one rhizome with 6–7 shoots and corresponding roots, and water were collected at each donor site (CO_2_ and Reference) and quickly transported (less than 5 minutes) to an intermediate incubation location, halfway of donor sites, where all incubations took place at 2 m depth. Plants were gently cleaned from sediment and epiphytes and placed into the incubation chambers with water from their own collection site (*in situ* incubations) or from the other site (transplant incubations).

For each type of incubation (*in situ* or transplants) two runs were conducted, one at midday (around 12:00 h) and one in the afternoon (around 16:00 h). At each run, four incubation chambers from each site were incubated together and thus they were exposed to the same irradiance. Light measurements (Licor sensor) were done at the incubation location along the incubation time. No significant differences were found between the midday (*t*-test, p = 0.7) or the afternoon (p = 0.2) irradiances during the two consecutive days of incubations.

**Figure 5:**
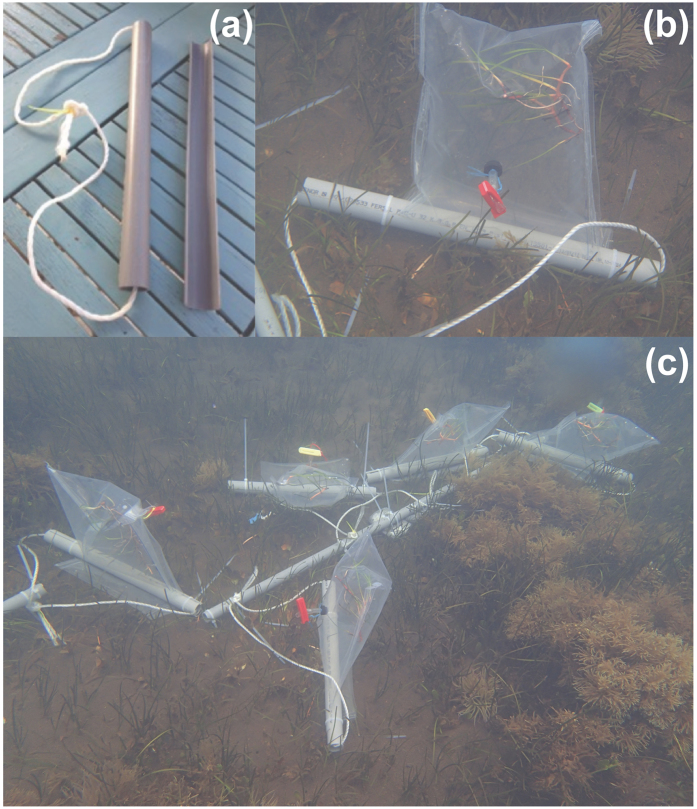
Incubation chambers. (**a**) Detail of closing system, (**b**) deployed incubation chamber and (**c**) running set of incubations.

Incubation chambers consisted of a gas-tight polyethylene plastic bag (30 cm long, 20 cm wide, 2.5 L approx.) with one sampling port to withdraw water samples (Fig. 5b). Chambers were sealed with a clamp made of a polyvinyl chloride (PVC) half-cylinder adjustable to a PVC rigid bar (Fig. 5a). Chambers were mounted and fixed in a steel bar and incubated together at 2 m depth (Fig. 5c). The positive buoyancy and flexible nature of the plastic bags allowed propagation of external turbulence to the interior of the chamber to prevent too thick surface boundary layers^55^. Incubation time ranged between 1–2 h to avoid within chamber O_2_ saturation effects^56^. Water samples were taken at the beginning and end of each incubation and rapidly preserved for oxygen determination (see below). Plants were weighted and measured for NPP normalization and a shoot subsample was collected for gene expression analysis (see below).

In Ria Formosa, *in situ* incubations (n = 64) were conducted at 2 m depth to measure the productivity of *C. nodosa* to varying CO_2_ concentrations. Seawater used in the incubations was collected from the field and bubbled with air at different concentrations of CO_2_, ranging from ~250 up to ~1500 μatm (pCO_2_) (i.e. ~2000 to ~2400 μmol Kg SW^−1^ DIC and 8.39 to 7.70 pH (NBS scale)), thus covering the range found in Vulcano and the predicted CO_2_ scenarios for the end of the century^1^ without modifying any other seawater parameters. Water and biomass samples were processed as in Vulcano.

### Gene expression

#### Material collection, RNA extraction and cDNA preparation

One shoot from three randomly chosen incubation chambers was collected at the end of both *in situ* and transplants incubations. Apical (youngest) shoots were avoided and only middle parts of the youngest fully mature leaves (2° or 3° rank leaves) of intermediate shoots were selected to standardize leaf developmental stages. Immediately after collection, leaves were blotted dry with tissue paper and preserved in RNAlater© (Ambion, Life technologies) to protect RNA from degradation. The whole procedure, from collection to fixing, lasted less than 5 minutes. After one night at 4 °C, samples were stored at −20 °C until RNA extraction. RNA was extracted from *C. nodosa* leaves with the Aurum™ Total RNA Mini Kit (BIO-RAD) following manufacturer’s instructions. The full protocol for RNA extraction and cDNA preparation is described in Appendix 2, Supplementary Information.

#### Gene selection and RT-qPCR

Reverse Transcription-quantitative Polymerase Chain Reaction (RT-qPCR) analysis was performed to investigate differences in expression levels of genes belonging to major pathways affected by changes in CO_2_ availability (i.e. photosynthesis, carbon assimilation pathways) that can be directly correlated with plant productivity (psaJ, psaC, psbA, psbD, LHCA1, FD, rbcL, ATPA, PEPC, SUS and BCA). Additionally, some studies have related high-CO_2_ environments with antioxidant and oxidative stress responses in land plants, and more recently in other seagrass species growing close to natural volcanic CO_2_ vents^22^, so a number of genes involved in free-radical detoxification and oxidative-stress response were also targeted (SOD, CAT, APX7, APX6, LBP, GSH-S and GR). Finally, since the presence of metals and toxic trace elements has been shown in fluid emissions near some vents, also two genes related to metal detoxification (MTP and MT) were selected. Specific primer pairs were established and tested for 8 putative reference genes (RGs) and 20 genes of interest (GOIs). (The full RT-qPCR protocol and gene description for selected genes are available in Appendix 2 and Supplementary Table S10.

### Net plant productivity

Net plant productivity (NPP) was estimated following the methodology described in ref. 56. Initial and final water samples were collected from each chamber with plastic syringes (100 mL) at the beginning and end of the incubations, respectively. Once collected, syringes were quickly transported to shore for sample fixation (2 m away from the incubation site). For oxygen fixation, Exetainer vials (Labco Limited) (12 mL) (n = 3) were filled with water from each syringe. Dissolved oxygen concentration (O_2_) was determined spectrophotometrically by the modified Winkler method according to the protocol described in ref. 57. Net plant productivity (NPP, μmol O_2_ g DW^−1^ h^−1^) rates were calculated by the difference between final and initial oxygen concentration normalized by the incubation time, the volume of water and the biomass incubated.

### Statistics

Normality (Shapiro) and homoscedasticity (Levene) tests were run to test parametric assumptions. Differences in seawater parameters between sites in Vulcano were tested with paired *t*-tests.

For gene expression, multivariate differences in gene expression among different treatments were tested by an analysis of similarity (ANOSIM), with Primer 6 (v.6.1.12). To inspect the significant gene regulation in the different conditions (*in situ* incubations and transplants) relative to controls, we used the method developed in REST 2009 Software version 2.0.13^58^. REST 2009 Software provide a means for determining the mean output and a p value for the likelihood of upregulation or downregulation using a hypothesis test. Statistical significance was set at p < 0.05.

To investigate the effect of CO_2_ on productivity (NPP) of *C. nodosa* plants long- (*in situ* incubations) and short-term (transplants incubations) exposed to high CO_2_ water from CO_2_ vents, an ANCOVA analysis considering two factors, the “origin” of the plants (plants from CO_2_ or Reference site) and the “water” used in the incubation (water from CO_2_ or Reference site), and “irradiance” (as a continuous covariate) was performed in order to account for any potential effect of irradiance on NPP. ANCOVA analysis indicated a highly significant main effect of the “water” (p = 0.002) whereas the effects of “irradiance” or “origin” were not significant. A significant three-way (irradiance, origin, water) interaction was also revealed (p = 0.03), but the post-hoc tests did not provide a clear interpretation of the occurrence of any other relevant processes, i.e. it was not clear what explanatory factors needed to be retained in the model. We then tested for a model simplification using an Akaike’s information criterion (AIC) test among 3-way and 2-way factorial and additive possible ANCOVA and ANOVA models. The 2-way ANOVA (two fixed crossed factors) with “origin” and “water” as explanatory factors produced the lowest AIC value, that is, the best fit and explanatory power indicating that a simplification of the ANCOVA model was justified^59^. We retained the two-way ANOVA (two fixed crossed factors) as the most adequate model to interpret the NPP results. Vulcano NPP data were log-transformed to meet parametric assumptions.

Likewise, at Ria Formosa the incubations were carried out along the day with changing irradiances. A multiple regression analysis including irradiance and DIC as explanatory variables was performed to investigate the effect of CO_2_ on productivity (NPP) of *C. nodosa* plants. The explanatory power of DIC was highly significant (p = 0.01) as opposed to irradiance (p = 0.2) and the best AIC fit was obtained with DIC as single explanatory variable. For this reason we performed a linear regression with DIC as single explanatory variable.

To analyse the combined response of gene expression patterns and NPP on plants from *in situ* and transplant incubations, a Principal Component Analysis (PCA) and a Cluster analysis were also performed with the software PAST (v.3.03). To link gene expression to productivity a Spearman’s Rank correlation analysis was conducted between the NPP and expression of each gene measured (n = 20 genes) considering the mean gene expression of each incubation type (n = 4 ranks, i.e. *in situ* and transplant incubations for both Reference and CO_2_ plants). Unless otherwise indicated, data were analysed using R^60^.

## Additional Information

**How to cite this article**: Olivé, I. *et al*. Linking gene expression to productivity to unravel long- and short-term responses of seagrasses exposed to CO_2_ in volcanic vents. *Sci. Rep.* **7**, 42278; doi: 10.1038/srep42278 (2017).

**Publisher's note:** Springer Nature remains neutral with regard to jurisdictional claims in published maps and institutional affiliations.

## Supplementary Material

Supplementary Information

## Figures and Tables

**Table 1:** Irradiance and seawater physico-chemical parameters during the incubations.

	Vulcano (Italy)		Ria Formosa (Portugal)
Irradiance (μmol quanta m^−2^ s^−1^)	Midday: 1137 ± 65		517–1326
	Afternoon: 539 ± 107		
	**Reference site**	**CO**_**2**_ **site**	
S	37.5 ± 0.0	37.5 ± 0.0	36.3 ± 0.3
T (°C)	19.8 ± 0.5	20.3 ± 1.0 (20.2 ± 0.8)	25.7 ± 1.6
pH (NBS)	8.179 ± 0.058	7.985 ± 0.088 (7.896 ± 0.193)	7.706–8.387
TA (μmol · kg SW^−1^)	2545 ± 7	2577 ± 1 (2579 ± 12)	2466 ± 77
DIC (μmol · kg SW^−1^)	2244 ± 40	2377 ± 37 (2417 ± 86)	2018–2373
pCO_2_ (μatm)	427 ± 68	737 ± 158 (1004 ± 549)	245–1465
CO_2_ (μmol · kg SW^−1^)	14 ± 2	23 ± 6 (32 ± 17)	7–38
HCO_3_^−^ (μmol · kg SW^−1^)	2012 ± 61	2197 ± 63 (2250 ± 117)	1685–2227
CO_3_^2−^ (μmol · kg SW^−1^)	218 ± 23	156 ± 32 (136 ± 49)	98–359

Data are mean ± sd or min–max values recorded in the water. Irradiance was measured 0.5 m above the incubation chambers. Data in parenthesis include one extreme high value recorded in the CO_2_ site in Vulcano.

**Table 2:** List of reference genes (RGs) and genes of interest (GOIs) analysed in *Cymodocea nodosa*.

Gene symbol	Protein name	Gene Ontology
Reference genes (RGs)		
18S	Ribosomal RNA 18S	Translation
eIF4A	Eukaryotic initiation factor 4A	Translation/Protein biosynthesis
GAPDH	Glyceraldehyde-3-phosphate dehydrogenase	Glycolysis
UBI	Ubiquitin	Ubiquitin-dependent protein catabolic process
Genes of interest (GOIs)		
psaJ	Photosystem I reaction center subunit IX	Photosynthesis
psaC	Photosystem I iron-sulfur center	Photosynthesis
psbA	Photosystem II protein D1	Photosynthesis
psbD	Photosystem II protein D2	Photosynthesis
LHCA1	Photosystem I Light Harvesting Complex gene 1	Photosynthesis
FD	Ferredoxin, chloroplastic	Electron transport chain
rbcL	RuBisCO large subunit	Carbon dioxide fixation
ATPA	ATP synthase subunit alpha	ATP biosynthetic process
PEPC	Phosphoenolpyruvate carboxylase	Carbon dioxide fixation
SUS	Sucrose synthase	Sucrose metabolic process
BCA	Beta carbonic anhydrase	Carbon utilization
SOD	Copper/zinc superoxide dismutase	Response to oxidative stress
CAT	Catalase	Response to oxidative stress
APX7	L-ascorbate peroxidase 7, chloroplastic	Response to oxidative stress
APX6	L-ascorbate peroxidase 6	Response to oxidative stress
LBP	Luminal binding protein	Response to oxidative stress
GSH-S	Glutathione synthase	Glutathione biosynthetic process
GR	Glutathione reductase	Glutathione metabolic process
MTP	Metal tolerance protein	Ion transport
MT	Metallothionein	Cellular metal ion homeostasis

Gene names, gene encoding protein names and gene ontology are given.
