# Supplementary material for: Linking gene expression to productivity to unravel long- and short-term responses of seagrasses exposed to CO2 in volcanic vents
Source: Sci Rep. 2017 Feb 13;7:42278. doi: 10.1038/srep42278 (PMC5304229; doi:10.1038/srep42278)
Supplement: Supplementary Information [file srep42278-s1.pdf]

ELECTRONIC SUPPLEMENTARY INFORMATION (SI)

TITLE

Linking gene expression to productivity to unravel long- and short-term responses of seagrasses exposed to CO_2 in volcanic vents

AUTHORS

Irene Olivé, João Silva, Chiara Lauritano, Monya M. Costa, Miriam Ruocco, Gabriele Procaccini, Rui Santos

APPENDIX 1

Supplementary information on Results. Selection, stability and ranking of reference genes in *Cymodocea nodosa*. Results of two-way ANOVA test on productivity of *C. nodosa*.

Table S1. Selection of reference genes in *Cymodocea nodosa* based on the BestKeeper approach [1]. Lowest standard deviation (SD) is underlined. *n*, number of plant samples in the tested conditions; GM, geometric mean of C_T-value; AM, arithmetic mean of C_T-value; Min and Max, the extreme values of C_T; SD, standard deviation of C_T; CV, coefficient of variance as percentage.

	18S	eIF4A	GAPDH	UBI
n	12	12	12	12
GM [CT]	22.53	27.83	26.05	27.31
AM [CT]	22.54	27.83	26.06	27.31
min [CT]	21.43	26.91	25.12	26.44
max [CT]	23.38	28.72	27.26	28.23
SD [± CT]	0.50	0.35	0.55	0.46
CV [% CT]	2.23	1.28	2.12	1.67

Table S2. Expression stability of candidate RGs as calculated by geNorm [2] in *Cymodocea nodosa*. Best candidate genes are underlined.

**Gene name**	**Average expression stability (*M*)**
eIF4A	0.60
GAPDH	0.60
18S	0.71
UBI	0.83

Table S3. Expression stability of candidate RGs as calculated by NormFinder [3] in *Cymodocea nodosa*. Best candidate genes are underlined.

**Gene name**	**Stability value**	**Standard error**
eIF4A	0.07	0.10
18S	0.25	0.07
GAPDH	0.25	0.07
UBI	0.31	0.08

Table S4. Ranking of the reference gene candidates by BestKeeper, geNorm, and NormFinder. The three best RGs selected for RT-qPCR assays are highlighted in bold. S.D.: standard deviation; Stab. Val.: stability value; *M*: average expression stability.

**Bestkeeper**	**NormFinder**	**geNorm**
(S.D.)	(Stab. Val.)	(*M*)
**eIF4A** (0.35)	**eIF4A** (0.07)	**eIF4A** (0.60)
UBI (0.46)	**18S** (0.25)	**GAPDH** (0.60)
18S (0.50)	GAPDH (0.25)	18S (0.71)
GAPDH (0.55)	UBI (0.31)	UBI (0.83)

Table S5. Results of ANOSIM multivariate gene expression analysis. *In situ* incubations indicate gene expression of CO_2 plants incubated in CO_2 water with respect to Reference plants incubated in Reference water. Transplant incubations (Reference plants) indicate gene expression of Reference plants incubated in CO_2 water with respect to Reference plants incubated in Reference water. Transplant incubations (CO_2 plants) indicate gene expression of CO_2 plants incubated in Reference water with respect to CO_2 plants incubated in CO_2 water.

	Global Test		
	**Sample statistic (Global R)**	**Significance level**	**Average dissimilarity**
In situ incubations	0.37	10%	10.39
Transplant incubations (Reference plants)	-0.074	70%	8.08
Transplant incubations (CO_2 plants)	0.37	20%	6.51

Table S6. Relative expression report for RT-qPCR experiments obtained with REST 2009 [4]. Expression values (with Standard error, Std. Error) and P(H1) (Probability of alternate hypothesis that difference between sample and control groups is due only to chance) are reported for each gene. (a) *In situ* incubations indicate relative gene expression of CO_2 plants incubated in CO_2 water with respect to Reference plants incubated in Reference water. (b) Transplant incubations (Reference plants) indicate relative gene expression of Reference plants incubated in CO_2 water with respect to Reference plants incubated in Reference water. (c) Transplant incubations (CO_2 plants) indicate relative gene expression of CO_2 plants incubated in Reference water with respect to CO_2 plants incubated in CO_2 water. Underlined values indicate P(H1) < 0.1.

**(a) In situ incubations**			
**Gene**	**Expression**	**Std. Error**	**P(H1)**
psaJ	-1.2235	0.5444	0.351
psaC	-1.5637	0.3760	0.268
psbA	-3.1933	0.2377	0.000 ***
psbD	-1.1583	0.4907	0.330
LHCA1	-6.1860	0.1217	0.000 ***
FD	-2.1205	0.3018	0.058
rbcL	-1.7125	0.2872	0.037 *
ATPA	-1.0020	0.5949	0.461
PEPC	-4.6031	0.2156	0.058
SUS	-1.7949	0.2028	0.000 ***
BCA	-4.3563	0.1641	0.000 ***
SOD	-2.6559	0.2845	0.000 ***
CAT	-2.1268	0.2985	0.107
APX7	-1.7292	0.2444	0.000 ***
APX6	-2.0806	0.3129	0.000 ***
LBP	-1.4587	0.3902	0.000 ***
GSH-S	-2.2658	0.2362	0.000 ***
GR	-3.1383	0.2030	0.037 *
MTP	-2.7702	0.1626	0.000 ***
MT	-3.4187	0.2834	0.000 ***

(b) Transplant incubations (Reference plants)			
**Gene**	**Expression**	**Std. Error**	**P(H1)**
psaJ	-1.6625	0.6732	0.602
psaC	-2.0596	0.3616	0.212
psbA	-1.5462	0.2615	0.192
psbD	-1.6521	0.9551	0.704
LHCA1	1.0088	0.6195	0.969
FD	1.2004	1.1975	0.793
rbcL	-1.5319	0.4360	0.358
ATPA	-1.5760	0.6649	0.528
PEPC	-2.3135	0.1910	0.000 ***
SUS	-1.1906	0.7741	0.778
BCA	-1.2465	0.3835	0.819
SOD	1.2143	0.5284	0.713
CAT	-1.1480	0.4479	1.000
APX7	1.1068	0.6279	0.704
APX6	-1.4624	0.3958	0.389
LBP	-1.2174	0.9836	0.894
GSH-S	-1.8769	0.2877	0.212
GR	-1.7806	0.2851	0.000 ***
MTP	-1.0744	0.9455	0.894
MT	-1.0346	0.3725	0.894

(c) Transplant incubations (CO_2 plants)			
**Gene**	**Expression**	**Std. Error**	**P(H1)**
psaJ	1.5063	0.5530	0.000 ***
psaC	-1.7183	0.2825	0.127
psbA	2.9783	2.2956	0.096
psbD	2.0434	0.9608	0.190
LHCA1	4.4506	2.5997	0.074
FD	1.6250	1.6696	0.529
rbcL	1.3850	0.6230	0.000 ***
ATPA	1.1772	0.7049	0.704
PEPC	2.1562	2.1473	0.497
SUS	2.2300	0.8250	0.095
BCA	2.6931	1.9187	0.096
SOD	2.4607	1.7860	0.101
CAT	1.9327	1.0148	0.000 ***
APX7	1.7995	0.6850	0.095
APX6	1.5200	0.7488	0.000 ***
LBP	1.6729	0.7234	0.601
GSH-S	2.1214	0.9188	0.000 ***
GR	3.7192	2.3486	0.096
MTP	3.6992	1.5363	0.075
MT	5.8535	5.7004	0.101

Table S7. Results of two-way ANOVA test (fixed crossed factors) to assess the effects of the "origin" of the plants (plants from CO_2 or reference site) and the "water" used in the incubations (water from CO_2 or reference site) of *Cymodocea nodosa* plants in Vulcano.

	**Df**	**Sum Sq**	**Mean Sq**	**F value**	**Pr(>F)**	
Origin	1	0.0796	0.0796	1.484	0.23457	
Water	1	0.6161	0.6161	11.481	0.00233	**
Origin x Water	1	0.1448	0.1448	2.698	0.113	
Residuals	25	1.3415	0.0537			

Table S8. Results of Principal Components Analysis (PCA) conducted considering the combined contributions of all GOIs and NPP. Percent variation explained by the first three axes and individual contribution of NPP and targeted genes are indicated.

PC	Eigenvalue	%variance	
1	9.2212	66.465	
2	3.5521	25.603	
3	1.1004	7.9316	
	PC 1	PC 2	PC 3
NPP	0.1737	0.0222	0.3656
psaJ	0.0461	0.0163	-0.0246
psaC	0.0110	0.0999	0.5127
psbA	0.2576	0.0840	0.0199
psbD	0.0188	0.0812	-0.2113
LHCA1	0.2701	0.4156	-0.1125
FD	0.0487	0.2814	0.1668
rbcL	0.0476	0.1879	0.0989
ATPA	-0.0107	0.0423	0.0005
PEPC	0.3636	0.0326	0.2246
SUS	0.0558	0.2356	-0.1749
BCA	0.1960	0.3233	0.1975
SOD	0.3551	-0.1987	0.0293
CAT	0.2353	0.0265	-0.3159
APX7	0.0029	0.3011	-0.0525
APX6	0.1043	0.1223	0.2124
LBP	-0.0125	0.2373	0.0102
GSH-S	0.2933	-0.1526	-0.0730
GR	0.4466	-0.3454	0.1849
MTP	0.0836	0.4169	-0.1732
MT	0.4055	-0.0834	-0.4116

Table S9. Spearman's rank correlation between Net plant productivity (NPP) and mean gene expression from the incubations of *Cymodocea nodosa* plants in Vulcano.

	Spearman coefficient (ρ)	p-value	
NPP	1	0	
psaJ	0.49	0.0064	**
psaC	0.3	0.1099	
psbA	0.49	0.0064	**
psbD	-0.06	0.738	
LHCA1	0.49	0.0064	**
FD	0.29	0.132	
rbcL	0.29	0.132	
ATPA	-0.15	0.4266	
PEPC	0.59	0.0007	***
SUS	-0.27	0.1525	
BCA	0.29	0.132	
SOD	0.59	0.0007	***
CAT	0.29	0.1236	
APX7	-0.07	0.7143	
APX6	0.29	0.132	
LBP	-0.07	0.7143	
GSH-S	0.39	0.0356	*
GR	0.59	0.0007	***
MTP	-0.07	0.7143	
MT	0.29	0.1236	

APPENDIX 2

Supplementary information on Material and Methods. Gene expression protocol and information on Reference genes (RGs) and Genes of interest (GOIs).

RNA extraction and cDNA preparation

RNA was extracted from *C. nodosa* leaves with the Aurum™ Total RNA Mini Kit (BIO-RAD) following manufacturer's instructions and retro-transcribed in cDNA with the iScript™ cDNA synthesis kit (BIO-RAD), according to standard protocol. RNA quantity and purity were assured by Nano-Drop (ND-1000 UV-Vis spectrophotometer; NanoDrop Technologies) and by gel electrophoresis. The 260/280 nm and 260/230 nm ratios were always about 2.0, while gel electrophoresis showed intact RNA, with sharp ribosomal bands. Total RNA (500 ng) was retro-transcribed in cDNA with the iScript™ cDNA synthesis kit (BIO-RAD), according to standard protocol.

Gene selection and RT-qPCR

A Reverse Transcription -quantitative Polymerase Chain Reaction (RT-qPCR) analysis was performed in order to investigate differences in expression levels of genes belonging to pathways potentially affected by changes in CO_2 availability (photosynthesis, carbon utilization, free-radical detoxification and oxidative-stress response, and metal detoxification) (Supplementary Table S10).

Given the lack of genomic (or transcriptomic) resources available for this species, specific primers for putative reference genes (RGs) and genes of interest (GOIs) were designed considering the alignment of conserved regions in other monocotyledons such as *Posidonia oceanica*, *Zostera marina*, *Oryza sativa*, *Zea mays*. Sequences were

identified using the search function in the seagrass EST database Dr. Zompo (http://drzompo.uni-muenster.de) or the generic online database GenBank (http://www.ncbi.nlm.nih.gov/genbank).

Alignments were performed with ClustalW embedded in BioEdit v. 7.0.5.3 [5]. Primers were designed with the primer analysis software Primer3 v. 0.4.0 [6,7] and Gene Runner v. 3.05 (Hasting Software, Inc.). Primers' selection conditions included primer length (18-23 bp), T_m (60°C), GC content (≥50%) and product size (130 to 230 bp). Primers for *SUS* and *rbcL* were designed from the known *C. nodosa* sequences available in the GenBank database (Acc. no. AM292651.2 and U80688.1, respectively). In some cases primer pairs established in previous works for closely related species were used (see references in Table S10).

To determine the specificity of the amplification, designed primer pairs were first tested in PCR, according to the reaction conditions detailed in [8]. Amplified PCR products were then analyzed by 1.5% agarose gel electrophoresis in TBE buffer. The resulting bands were excised from the gel and extracted with the GenElute™ Gel Extraction Kit (SIGMA). Sequence reactions were obtained with the BigDye Terminator Cycle Sequencing technology (Applied Biosystems, Foster City, CA), purified in automation using the Agencourt CleanSEQ Dye terminator removal Kit (Agencourt Bioscience Corporation, 500 Cummins Center, Suite 2450, Beverly MA 01915 - USA) and a robotic station Biomek FX (Beckman Coulter, Fullerton, CA). Products were analyzed on an Automated Capillary Electrophoresis Sequencer 3730 DNA Analyzer (Applied Biosystems). The identity of each sequence was confirmed using the blastn function. All obtained *C. nodosa* sequences are deposited in GenBank under the accession numbers shown in Table S10.

We set primers for 8 RGs and 20 GOIs, but 4 RGs (*L31*, *UBC13*, *EF1A* and *TBP*) were discarded due to double or no amplifications [8]

RT-qPCR was performed in MicroAmp Optical 384-Well reaction plate (Applied Biosystems) with Optical Adhesive Covers (Applied Biosystems) on a Viia7 Real Time PCR System (Applied Biosystems), using Sybr Green as fluorescence detection chemistry. The PCR consisted of 5 µL Fast SYBR® Green Master Mix (Applied Biosystems), 1 µL 1:50-diluted cDNA template, and 0.7 pmol μL^{-1} of each primer, in a total reaction volume of 10 µL. Thermal profile was as follows: 20 sec at 95°C, 40 cycles of 1 sec at 95°C and 20 sec at 60°C. For determining the specificity of the reaction, the melting curve of each product from 60 to 95°C was also assessed. All RT-qPCR reactions were carried out in triplicate and each assay included three no-template negative controls (NTC) for each primer pair [9]. The technical variation among the triplicates was checked and individual outliers were excluded when the SD was more than 0.3.

PCR efficiencies for all primer pairs have been calculated from the slopes of standard curves of the threshold cycle (C_T) *vs.* cDNA concentration, with the equation $E = 10^{-1/slope}-1$. Primer's sequences, percent efficiencies (E) and regression coefficients (R^2) of RGs and GOIs are reported in Table S10. All $E \geq 84\%$ and all R^2 were ≥ 0.97. To normalize target gene-expression data, three different algorithms BestKeeper [1], geNorm [2] and NormFinder [3] were utilized to identify the best RGs in our experimental conditions, among the four candidate RGs selected for amplification: the Eukaryotic initiation factor 4A (eIF4A), the Glyceraldehyde 3-phosphate dehydrogenase (GAPDH), the 18S ribosomal RNA and the Ubiquitin (UBI). All RGs belong to different functional classes in order to reduce the possibility that they might be co-regulated. For the relative quantification of RT-qPCR data, we used REST-

MCS© (Relative Expression Software Tool) [4]. The mathematical model used is based on the correction for exact PCR efficiencies and the mean crossing point deviation between sample group(s) and control group(s), and it allows the use of multiple reference genes for the normalization of target gene expression levels [10].

TABLES

Table S10. List of reference genes (RGs) and genes of interest (GOIs) in *Cymodocea nodosa* assessed using RT-qPCR. Gene symbol and protein name, GenBank accession number, primer sequence, reference, amplicon size (S, base pair), percent of efficiency (*E*), correlation coefficient (R^2) and Swiss-Prot best hit with corresponding *E*-value are given.

Gene symbol	Protein name	GenBank	Primer Sequences 5' ->3'	Reference	S	%*E*	R^2	Swiss-Prot best hit	E-Value
Reference genes (RGs)									
18S	Ribosomal RNA 18S	KT200607	F:AACGAGACCTCAGCCTGCTA R:AAGATTACCCAAGCCTGTCG	8	200	100	0.97	-	-
eIF4A	Eukaryotic initiation factor 4A	KT200591	F:TTCTGCAAGGGTCTTGACGT R:TCACACCCAAGTAGTCACCAAG	11	194	100	0.99	sp|Q40466.1|IF413_TOBAC	2e-37
GAPDH	Glyceraldehyde-3-phosphate dehydrogenase	KT200590	F:AGGTTCTTCCTGCTTTGAATG R:CTTCCTTGATTGCTGCCTTG	8	139	100	0.99	sp|P34783.1|G3P_ATRNU	5e-34
UBI	Ubiquitin	KT200589	F:CACCCTCGCTGACTACAACA R:TTTCTCAGCCTGACGACCTT	8	195	96	0.99	sp|P31753.2|RS27A_ASPOF	1e-18
Genes of interest (GOIs)									
psaJ	Photosystem I reaction center subunit IX	KT200587	F:GGTTTGGGTCTTTAGCAGGTC R:GAATGGGTGGGAGGAGAAAT	12	155	100	0.97	sp|Q6EW33.1|PSAJ_NYMAL	8e-20
psaC	Photosystem I iron-sulfurcenter	KT200593	F:TCTTGGGATGGGTGTAAAGC R:GGTTGTCTCATTCCATAAATACA		135	100	0.99	sp|P62090.2|PSAC_ARATH	4e-41
psbA	Photosystem II protein D1	KT200596	F:GACTGCAATTTTAGAGAGACGC R:CAGAAGTTGCAGTCAATAAGGTAG	12	137	97	0.99	sp|P27201.3|PSBA_LANPU	5e-71

	Harvesting Complex gene 1		R:AGTTCATCACCATCGCCTTC					B6_ARATH	
FD	Ferredoxin, chloroplastic	KT200600	F:ATGGTGAGCACCCCCTTC R:GGGTGACGAGCTTGACCTT	13	148	89	0.99	sp|Q43517.1|FER 1_SOLLC	3e-24
rbcL	RuBisCO large subunit	U80688.1	F:GCTGCCGAATCTTCTACTGG R:CACGTTGGTAACGGAACCTT	13	176	88	0.99	sp|P31196.2|RBL _NEMMU	1e-34
ATPA	ATP synthase subunit alpha	KT200595	F:TATCTGGCGATCTATTCAAT R:AACTCACGTAATCGTTGACC		226	88	0.98	sp|Q02848.1|ATP A_ANTSP	3e-28
PEPC	Phosphoenolpyruvate carboxylase	KT200592	F:AAGTTCCTACGCAGGCTTGA R:TGCCATCATTCTAGCCAACA		174	93	0.99	sp|P51059.1|CAP P2_MAIZE	5e-53
SUS	Sucrose synthase	AM29265 1.2	F:GATCCCAAGTTCAACATTGTCT R:CTCGCCATGGAGAAGATGAT		188	95	0.99	sp|Q41607.1|SUS 2_TULGE	3e-27
BCA	Beta carbonic anhydrase	KT200602	F:TACTGGGGTTTCAACCAGGA R:GGCTGTGACCAACGACTAAGA		153	99	0.99	sp|Q94CE3.1|BC A5_ARATH	1e-38
SOD	Copper/zinc superoxide dismutase	KT200606	F:GGAATGTCACAGCTGCAGAA R:CACCCGCATTTCCTGTAGTC		171	94	0.99	sp|O04996.3|SO DC_SOLCS	6e-32
CAT	Catalase	KT200585	F:CATCACATGCTGGGTTTCAC R:ACCGATCCTGGACATCTGAC	11	175	84	0.99	sp|P49317.1|CAT A3_NICPL	8e-33
APX7	L-ascorbate peroxidase 7, chloroplastic	KT200605	F:AAGAGGGGAGGAGCTAATGG R:GCTGGCAAGCTGAAACAAGT		150	93	0.99	sp|Q7XJ02.1|AP X7_ORYSJ	3e-34
APX6	L-ascorbate peroxidase 6	KT200603	F:TAATTGCTGTTGCCGGCTCT R:TCCTTTTTCACGGAAGCAAC		161	99	0.99	sp|Q8GY91.1|AP X6_ARATH	6e-24
LBP	Luminal binding protein	KT200583	F:ACCCGAGCTCGGTTTGAAGA R:ATTCTTGTGCTTCCACCAAC	11	137	90	0.99	sp|Q03685.1|BIP 5_TOBAC	2e-35
GSH-S	Glutathione synthase	KT200598	F:TAGGTTTCGCCAATTCTTGC R:AAGGGGTGGTTCTCCCAGAT	11	213	100	0.99	sp|P46416.3|GSH B_ARATH	4e-29
GR	Glutathione reductase	KT200599	F:TCCTCCAAGCTTAGTGCTTCA		134	100	0.99	sp|P48642.2|GSH	2e-37

			R:ACACAACCAGACGGTGTCAA					RC_ORYSJ	
MTP	Metal tolerance protein	KT200586	F:CTCGTTTCCTGAGGTTCTGC<br>R:TTTGCTGCTGTCATGGCTAC	11	183	91	0.99	sp|Q6Z7K5.1|MT<br>P3_ORYSJ	3e-26
MT	Metallothionein	KT200588	F:CATGTCGACCTGTGACAACTG<br>R:TATTAATGGCCACAGGTGCAG	11	195	96	0.99	sp|Q40256.1|MT<br>3_MUSAC	2e-22

REFERENCES

1 Pfaffl, M. W., Tichopad, A., Prgomet, C. & Neuvians, T. P. Determination of stable housekeeping genes, differentially regulated target genes and sample integrity: BestKeeper – Excel-based tool using pair-wise correlations. *Biotechnol. Lett.* **26**, 509-515 (2004).

2 Vandesompele, J. *et al.* Accurate normalization of real-time quantitative RT-PCR data by geometric averaging of multiple internal control genes. *Genome Biol.* **3**, doi:10.1186/gb-2002-3-7-20 research0034 (2002).

3 Andersen, C. L., Jensen, J. L. & Orntoft, T. F. Normalization of real-time quantitative reverse transcription-PCR data: a model-based variance estimation approach to identify genes suited for normalization, applied to bladder and colon cancer data sets. *Cancer Res.* **64**, 5245-5250 (2004).

4 Pfaffl, M. W., Horgan, G. W. & Dempfle, L. Relative expression software tool (REST (c)) for group-wise comparison and statistical analysis of relative expression results in real-time PCR. *Nucleic Acids Res.* **75**, 30 (2002).

5 Hall, T. A. BioEdit: a user friendly biological sequence alignment editor and analysis program for Windows 95/98/NT. *Nucleic Acids Symposium Series* **41**, 95-98 (1999).

6 Koressaar, T. & Remm, M. Enhancements and modifications of primer design program Primer3. *Bioinformatics* **23**, 1289-1291 (2007).

7 Untergrasser, A. *et al.* Primer3—new capabilities and interfaces. *Nucleic Acids Res.* **40**, e115 (2012).

8 Serra, I. A. *et al.* Reference genes assessment for the seagrass Posidonia oceanica in different salinity, pH and light conditions. *Mar. Biol.* **159**, 1269-1282, doi:10.1007/s00227-012-1907-8 (2012).

9 Bustin, S. A. *et al.* MIQE precis: practical implementation of minimum standard guidelines for fluorescence-based quantitative real-time PCR experiments. *BMC Mol. Biol.* **11**, 74, doi:10.1186/1471-2199-11-74 (2010).

10 Pfaffl, M. W. A new mathematical model for relative quantification in real-time RT–PCR. *Nucleic Acids Res.* **29**, e45-e45 (2001).

11 Lauritano, C. *et al.* Response of key stress-related genes of the seagrass *Posidonia oceanica* in the vicinity of submarine volcanic vents. *Biogeosciences* **12**, 4185-4194, doi:10.5194/bg-12-4185-2015 (2015).

12 Dattolo, E. *et al.* Response of the seagrass Posidonia oceanica to different light environments: Insights from a combined molecular and photo-physiological study. *Mar. Environ. Res.* **101**, 225-236, doi:10.1016/j.marenvres.2014.07.010 (2014).

13 Marín-Guirao, L., Ruiz, J. M., Dattolo, E., Garcia-Munoz, R. & Procaccini, G. Physiological and molecular evidence of differential short-term heat tolerance in Mediterranean seagrasses. *Sci. Rep.* **6**, 28615, doi:10.1038/srep28615 (2016).
